# Systemic inflammation is a determinant of outcomes of CD40 agonist–based therapy in pancreatic cancer patients

**DOI:** 10.1172/jci.insight.145389

**Published:** 2021-03-08

**Authors:** Max M. Wattenberg, Veronica M. Herrera, Michael A. Giannone, Whitney L. Gladney, Erica L. Carpenter, Gregory L. Beatty

**Affiliations:** 1 Division of Hematology-Oncology, Department of Medicine, and; 2 Abramson Cancer Center, Perelman School of Medicine, University of Pennsylvania, Philadelphia, Pennsylvania, USA.

**Keywords:** Immunology, Oncology, Cancer immunotherapy, Cellular immune response

## Abstract

Agonistic anti-CD40 monoclonal antibody (mAb) therapy in combination with chemotherapy (chemoimmunotherapy) shows promise for the treatment of pancreatic ductal adenocarcinoma (PDA). To gain insight into immunological mechanisms of response and resistance to chemoimmunotherapy, we analyzed blood samples from patients (*n* = 22) with advanced PDA treated with an anti-CD40 mAb (CP-870,893) in combination with gemcitabine. We found a stereotyped cellular response to chemoimmunotherapy characterized by transient B cell, CD56^+^CD11c^+^HLA-DR^+^CD141^+^ cell, and monocyte depletion and CD4^+^ T cell activation. However, these cellular pharmacodynamics did not associate with outcomes. In contrast, we identified an inflammatory network in the peripheral blood consisting of neutrophils, cytokines (IL-6 and IL-8), and acute phase reactants (C-reactive protein and serum amyloid A) that was associated with outcomes. Furthermore, monocytes from patients with elevated plasma IL-6 and IL-8 showed distinct transcriptional profiles, including upregulation of *CCR2* and *GAS6*, genes associated with regulation of leukocyte chemotaxis and response to inflammation. Patients with systemic inflammation, defined by neutrophil/lymphocyte ratio (NLR) greater than 3.1, had a shorter median overall survival (5.8 vs. 12.3 months) as compared with patients with NLR less than 3.1. Taken together, our findings identify systemic inflammation as a potential resistance mechanism to a CD40-based chemoimmunotherapy and suggest biomarkers for future studies.

## Graphical abstract

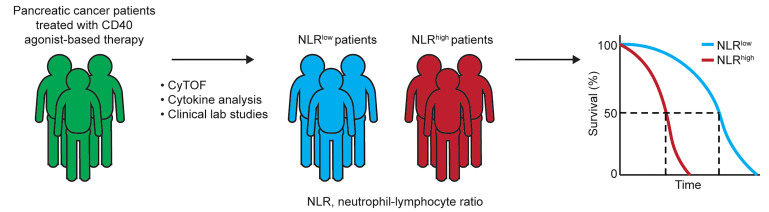

## Introduction

Pancreatic ductal adenocarcinoma (PDA) is a treatment-resistant cancer associated with significant morbidity and mortality (1, 2). Although current treatments are limited, deconvolution of PDA tumor biology has revealed novel therapeutic opportunities. To this end, the interaction between the immune system and PDA is now recognized to play a critical role in PDA biology and patient outcomes (3). The PDA tumor microenvironment (TME) is characterized by an inflammatory immune cell infiltrate, which is largely composed of immunosuppressive myeloid cells. Furthermore, the degree of myeloid cell infiltration is associated with reduced survival (4). In contrast, tumors from patients with PDA who are long-term survivors after surgery show high numbers of activated CD8^+^ T cells, suggesting that some patients with PDA develop productive antitumor immunity (5). These observations illustrate the dual role of the immune system in PDA. However, effective strategies for tipping the balance away from an immunosuppressive myeloid response and toward a productive antitumor T cell response remain elusive.

T cell immunotherapy, such as with monoclonal antibodies (mAbs) targeting cytotoxic T lymphocyte–associated protein 4 and programmed death 1/programmed death ligand 1 (PD-1/PD-L1) checkpoint proteins, has shown remarkable activity in patients with lung cancer, kidney cancer, and melanoma (6–8) but failed to improve outcomes for patients with PDA (9, 10). Deficient T cell priming due to abnormal dendritic cell (DC) frequency and function may limit the effectiveness of T cell immunotherapy in PDA (11, 12). As such, there is an emerging role for myeloid targeted immunotherapy, especially activation of DCs. For example, therapeutic activation of the tumor necrosis factor (TNF) superfamily member CD40, which is expressed by DC subsets, as well as other immune and nonimmune cells, has shown particular promise for the treatment of patients with PDA (13–16). We previously conducted a phase I clinical trial combining gemcitabine with an agonistic anti-CD40 mAb (CP-870,893) and found safety and evidence of clinical activity (17). Additionally, an ongoing phase Ib/II trial of a CD40 agonist (APX005M) in combination with gemcitabine and nab-paclitaxel with or without nivolumab showed an impressive overall response rate of 58% (18). However, not all patients respond to CD40 agonist–based therapy, and determinants of response and resistance remain ill-defined.

The mechanism of agonistic CD40 therapy has classically been considered “licensing” of DCs for T cell priming, leading to the activation of tumor-specific T cells (19). Supportive of this, in mouse models of PDA, DCs and CD4^+^ and CD8^+^ T cells are required for antitumor activity with a CD40 agonist in combination with chemotherapy (20, 21). However, systemic CD40 activation can also induce tumor regression via mechanisms not dependent on T cells, such as activation of tumoricidal macrophages and polarization of tumor-infiltrating myeloid cells, which sensitize PDA to chemotherapy (22, 23). These observations highlight the diverse antitumor actions of a CD40 agonist. Longitudinal analysis of a CD40 agonist in combination with chemotherapy for the treatment of patients with mesothelioma showed transient changes in frequency and phenotype of DCs and T cells in the peripheral blood (24). Additionally, in patients with PDA, anti-CD40 therapy is associated with depletion and activation of B cells (25, 26), However, beyond B cell pharmacodynamics, there is limited understanding of the cellular response to a CD40 agonist in patients with PDA.

Pretreatment patient-specific factors, including the presence of systemic inflammation, are known to be important determinants of outcomes in immunotherapy (27–30). Furthermore, PDA is often associated with development of a systemic inflammatory response (31), and several markers of systemic inflammation, including neutrophil/lymphocyte ratio (NLR), C-reactive protein (CRP), and serum amyloid A (SAA), are associated with poor outcomes in PDA (32–34). However, whether there is an interaction between systemic inflammation and treatment outcomes of a CD40 agonist remains unexplored in patients with PDA.

In this study, we use high-dimensional phenotyping, transcriptional analysis, and plasma cytokine analysis to evaluate immune contexture in the peripheral blood of patients with advanced PDA being treated with CD40-based chemoimmunotherapy. We find that although a stereotyped immune response occurs after treatment, cellular pharmacodynamics, including activation of T cells, are not associated with outcomes. Additionally, we show that systemic inflammation defines patients with distinct clinical and biological outcomes after treatment. Taken together, our findings provide novel insight into mechanisms of response and resistance to CD40-based therapy and identify potential biomarkers for future studies.

## Results

### Cellular response to CD40-based chemoimmunotherapy.

**Figure 1:**
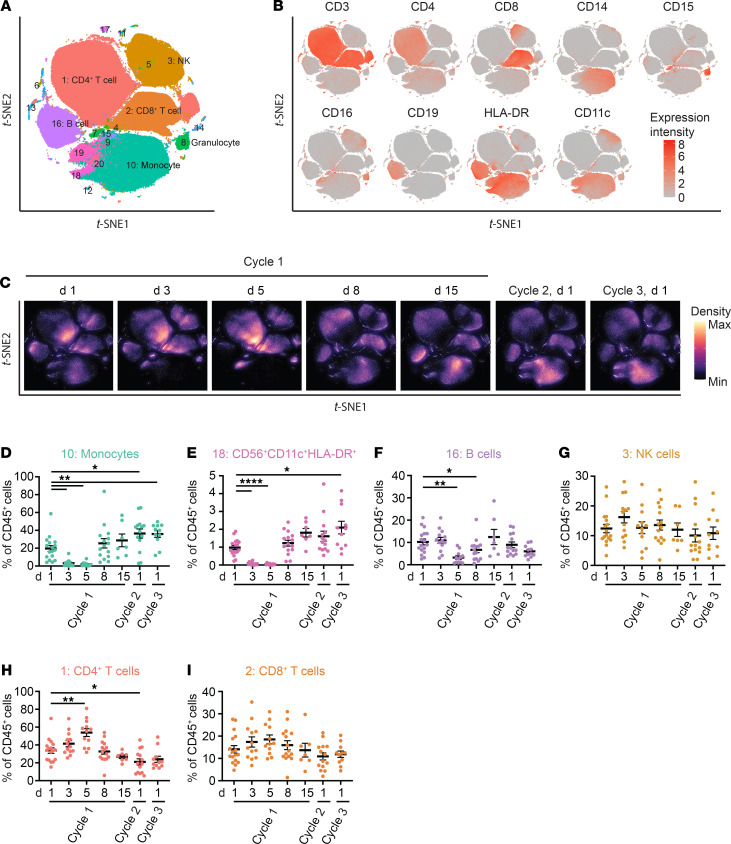
Cellular response to CD40 agonist–based chemoimmunotherapy. (**A**) After exclusion of doublets and dead cells and positive selection of CD45, samples, including patients and healthy volunteers (HVs), were downsampled to 5000 events and concatenated and FlowSOM clustering analysis was performed. (**B**) Marker expression level plots. (**C**) Density plots. (**D**–**I**) Quantification of cluster frequency. Mean ± SEM is shown. Day 1, *n* = 17; day 3, *n* = 13; day 5, *n* = 12; day 8, *n* = 15, day 15, *n* = 7, cycle 2, *n* = 14, cycle 3, *n* = 11. Mixed effects analysis with Dunnett’s multiple-comparison test was performed. *P < 0.05; **P < 0.01; ****P < 0.0001.

To assess the cellular response to a CD40 agonist in combination with chemotherapy (hereafter referred to as chemoimmunotherapy), we analyzed cryopreserved Ficoll-isolated PBMCs from patients (*n* = 17) with PDA treated with gemcitabine and an agonistic anti-CD40 mAb (Supplemental Figure 1A; supplemental material available online with this article; https://doi.org/10.1172/jci.insight.145389DS1). We used a mass cytometry–based (CyTOF) systems approach, which included a 37-marker metal-tagged antibody panel and unsupervised clustering (Phenograph, ref. 35) and metaclustering (FlowSOM, ref. 36), to define immune cell populations among all samples analyzed (Figure 1, A and B, and Supplemental Figure 1B). We then studied changes in immune cell metaclusters representing ≥1% of baseline PBMCs and saw dynamic remodeling of peripheral blood immune cell composition following chemoimmunotherapy (Figure 1C). After administration of gemcitabine on day 1 of treatment, depletion of monocytes (CD14^+^) was observed on days 3 and 5 with recovery to baseline levels by day 8. Additionally, monocytes were significantly increased at cycle 2, day 1, and cycle 3, day 1, as compared with baseline (Figure 1D). A minor CD14^+^ monocyte population, which expressed relatively higher levels of CD66a and CCR6 as compared with the major monocyte population, decreased in frequency on days 5 and 8 and then recovered to baseline levels thereafter (Supplemental Figure 1C). A CD56^+^CD11c^+^HLA-DR^+^CD141^+^ population also appeared to be influenced by gemcitabine administration and showed reduced frequencies on days 3 and 5, with recovery to baseline by day 8 (Figure 1E). Furthermore, anti-CD40 mAb therapy (administered on day 3) was associated with a transient decrease in B cells (CD19^+^) on day 5 with return to near baseline by day 8, as has been demonstrated previously (Figure 1F) (25). There was no change in natural killer (CD16^+^CD56^+^) cell frequency (Figure 1G). Granulocytes (CD14^–^CD15^+^CD66a^+^), which do not represent a major population in Ficoll-isolated PBMCs, did not change significantly over the course of treatment (Supplemental Figure 1D). Additionally, there was a relative increase in the frequency of CD4^+^ T cells among CD45^+^ cells but not CD8^+^ T cells at day 5 of treatment (Figure 1, H and I). Finally, a rare population expressing CD56, HLA-DR, CD11c, CD206, CD141, CD86, CX3CR1, and CCR6 was decreased on day 8 as compared with baseline (Supplemental Figure 1E).

### Treatment with CD40-based chemoimmunotherapy is associated with CD4^+^ T cell activation, which is uncoupled from outcomes.

**Figure 2:**
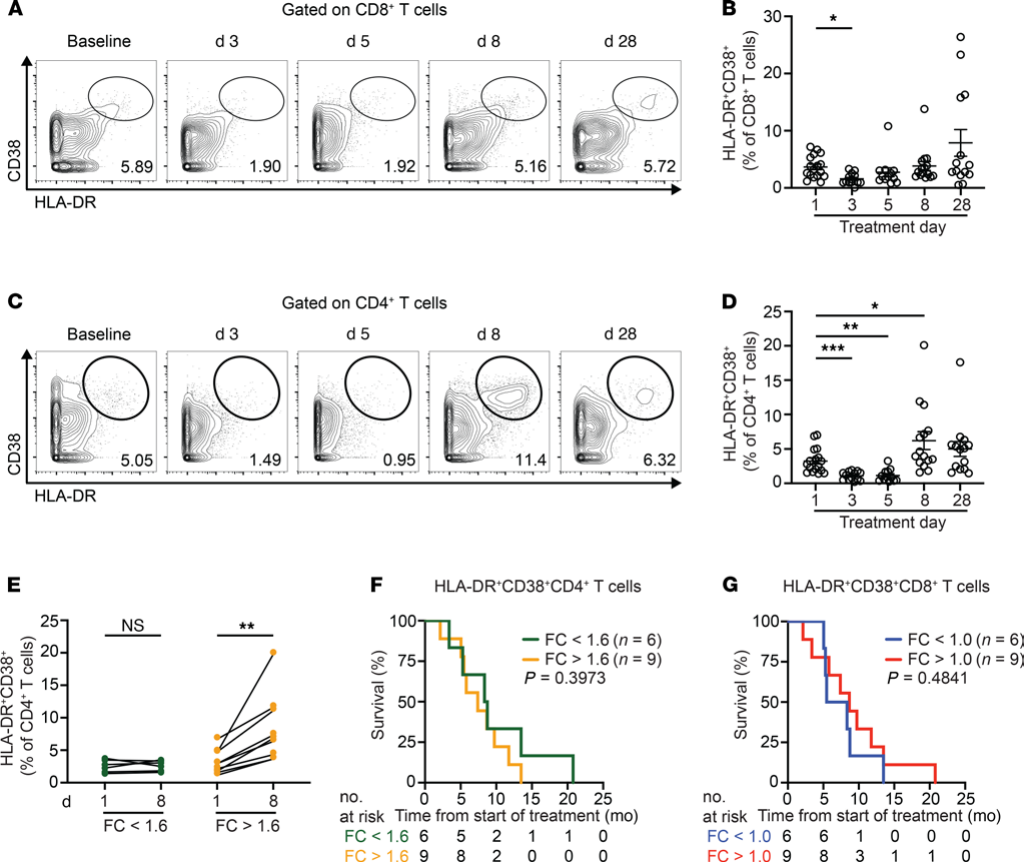
CD4^+^ T cell activation is not associated with outcomes of CD40 agonist–based chemoimmunotherapy. (**A**) Representative contour plots of HLA-DR^+^CD38^+^CD8^+^ T cells. (**B**) Quantification of HLA-DR^+^CD38^+^CD8^+^ T cells (as a percentage of CD8^+^ T cells). Mean ± SEM is shown. (**C**) Representative contour plots of HLA-DR^+^CD38^+^CD4^+^ T cells. (**D**) Quantification of HLA-DR^+^CD38^+^CD4^+^ T cells (as a percentage of CD4^+^ T cells). Shown is mean ± SEM. (**E**) Patients with stable (FC < 1.6) or increased (FC > 1.6) HLA-DR^+^CD38^+^CD4^+^ T cells between baseline and day 8. Wilcoxon’s matched pairs test was performed. (**F**) Overall survival (OS) was estimated by Kaplan-Meier methodology, and the log-rank test was used to determine significance. (**G**) Patients were dichotomized as having decreased (FC < 1.0) or increased (FC > 1.0) HLA-DR^+^CD38^+^CD8^+^ T cells between baseline and day 8, and Kaplan-Meier methodology and the log-rank test were used to compare OS. Day 1, *n* = 17; day 3, *n* = 13; day 5, *n* = 12; day 8, *n* = 15, day 28, *n* = 14. (**B** and **D**) Mixed effects analysis with Dunnett’s multiple-comparison test was performed. *P < 0.05; **P < 0.01; ***P < 0.001. FC, fold change.

Chemoimmunotherapy generates T cell–dependent antitumor immunity in mouse models of PDA (20, 21). Thus, we next asked whether chemoimmunotherapy influences T cell activation. To do this, we performed manual gating of the CyTOF data set to assess dual expression of CD38 and HLA-DR by T cells over the course of 1 cycle of treatment. Gemcitabine administration was followed by a transient decrease in HLA-DR^+^CD38^+^CD8^+^ T cells on day 3 of treatment, as compared with baseline (Figure 2, A and B). Four patients had an increase of CD8^+^ T cells expressing CD38 and HLA-DR at day 28 of treatment. The overall survival (OS) for these patients was 3.4, 5.1, 8.4, and 8.8 months. HLA-DR^+^CD38^+^CD4^+^ T cells significantly decreased on days 3 and 5 following gemcitabine administration and then significantly increased on day 8 following anti-CD40 mAb treatment, suggesting CD4^+^ T cell activation (Figure 2, C and D). Furthermore, we found heterogeneity in the CD4^+^ T cell response among patients (Figure 2E). However, there was no association between degree of CD4^+^ T cell activation and OS (Figure 2F). Similarly, when patients were dichotomized as having an increase or decrease in HLA-DR^+^CD38^+^CD8^+^ T cells at day 8 from baseline, there was no difference in OS among the 2 groups (Figure 2G).

### An inflammatory network is active in a subset of patients with advanced PDA.

**Figure 3:**
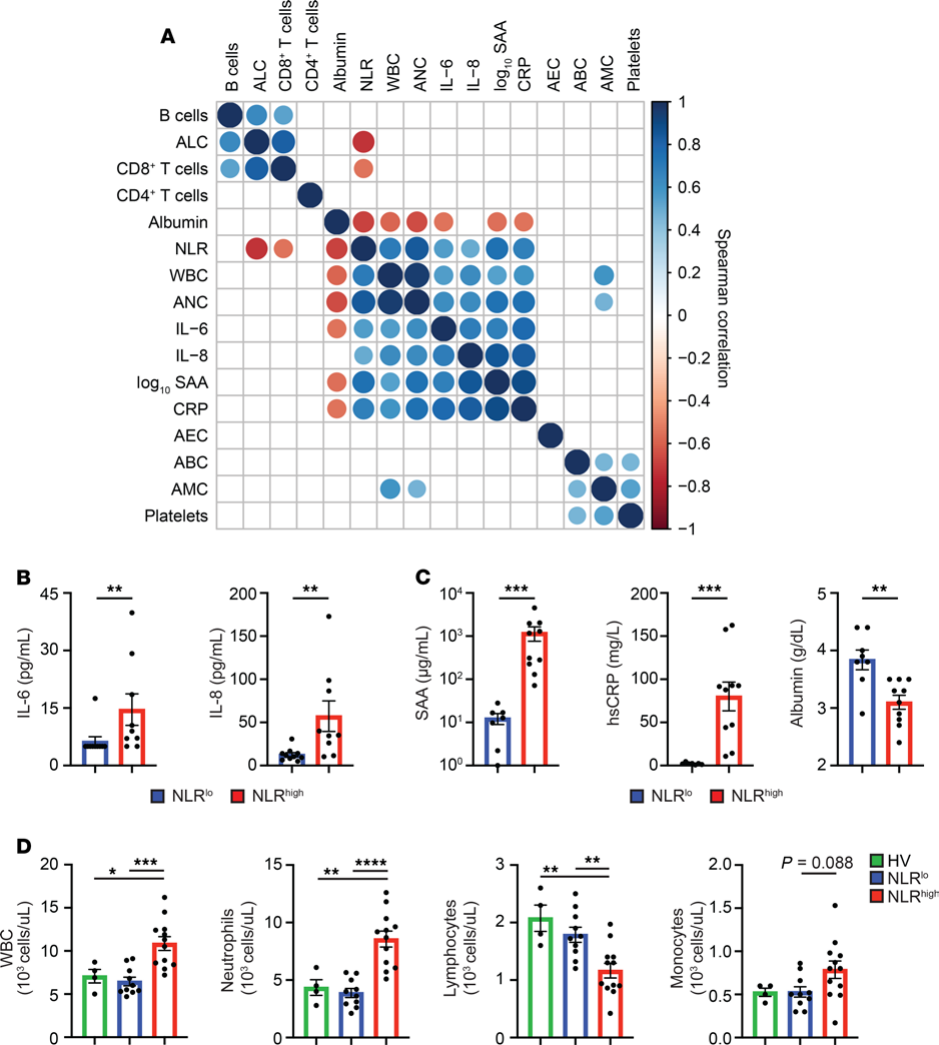
An inflammatory network is present in the blood of patients with PDA. (**A**) Correlation matrix displaying Spearman’s correlations among clinical blood counts, cytokines, and acute phase reactants of *n* = 22 patients. Correlations are shown when *P* < 0.05. Positive correlations are shown in blue and negative correlations are in red. (**B**) Quantification of inflammatory cytokines in patient plasma among NLR^lo^ (NLR < 3.1) and NLR^hi^ (NLR > 3.1) patients. (**C**) Quantification of acute phase reactants in patient plasma. (**D**) Quantification of clinical blood counts. Each dot represents an individual HV (green) or NLR^lo^ (blue) or NLR^hi^ patient (red). Mann-Whitney *U* tests (**B** and **C**) and 1-way ANOVA with Tukey’s multiple comparisons tests (**D**) were performed. *P < 0.05; **P < 0.01; ***P < 0.001, ****P < 0.0001. HV, healthy volunteer; SAA, serum amyloid A; hs, high-sensitivity; WBC, white blood cell count; ANC, absolute neutrophil count; NLR, neutrophil-lymphocyte ratio; ABC, absolute basophil count; AMC, absolute monocyte count; AEC, absolute eosinophil count; ALC, absolute lymphocyte count.

We also assessed pretreatment immune characteristics to define patient-specific determinants of responses to chemoimmunotherapy. We examined baseline levels of inflammatory cells, cytokines, and acute phase reactants in the peripheral blood of patients. CD4^+^ T cells, CD8^+^ T cells, and B cells were defined by manual gating of the CyTOF data set (Supplemental Figure 2). Although interpatient variability in levels of inflammatory markers was present, we found positive correlations among neutrophils, inflammatory cytokines (IL-6 and IL-8), and acute phase reactants (SAA and CRP), suggesting the presence of an inflammatory network (Figure 3A). Importantly, NLR, which is an established surrogate of systemic inflammation (37), showed a positive correlation with IL-6, IL-8, SAA, and CRP and a negative correlation with albumin, absolute lymphocyte count, and absolute CD8^+^ T cell count. These data demonstrate the presence of systemic inflammation in untreated patients with PDA and identify NLR as a measure of systemic inflammation in our patient cohort.

We next calculated pretreatment NLR using clinical blood counts and dichotomized patients using a previously established cutoff of 3.1 (32). Patients with NLR greater than 3.1 were defined as being systemically inflamed (NLR^hi^), and patients with NLR less than 3.1 were defined as being noninflamed (NLR^lo^). Classification of patients based on NLR identified biologically distinct groups based on pretreatment inflammatory factors. NLR^hi^ patients had significantly higher levels of IL-6, IL-8, SAA, and CRP and lower levels of albumin as compared with NLR^lo^ patients (Figure 3, B and C). Other cytokines associated with immune activation, including IL-2, IL-4, IL-5, IL-1β, IFN-γ, IL-10, IL-12, and TNF, were not found to be elevated at baseline (Supplemental Figure 3A). Using pretreatment clinical blood counts, we found NLR^hi^ patients to have significantly higher numbers of total white blood cells and neutrophils, numerically higher numbers of monocytes, and significantly lower numbers of lymphocytes as compared with NLR^lo^ patients and HVs (Figure 3D). Both NLR^hi^ and NLR^lo^ patients had similar numbers of eosinophils, basophils, and platelets (Supplemental Figure 3B). Using manually gated CyTOF data from the pretreatment time point, we detected lower absolute numbers of CD8^+^ T cells and NK cells in the peripheral blood of NLR^hi^ patients compared with NLR^lo^ patients, but this was not significant (Supplemental Figure 3C). In addition, we observed no significant difference in the percentage (of CD45^+^ cells) of B cells, T cells, NK cells, and DCs or the CD4^+^/CD8^+^ T cell ratio among NLR^hi^ and NLR^lo^ patients (Supplemental Figure 3, D and E). We also analyzed baseline cell clusters defined by FlowSOM that represented ≥1% of CD45^+^ cells among the 2 groups and found increased CD14^+^ monocytes in NLR^hi^ patients as compared with NLR^lo^ patients. However, this difference was not significant after corrections for multiple testing (Supplemental Figure 3F). Together, these data show the presence of an active inflammatory network in the peripheral blood of a subset of patients with PDA.

### Circulating monocytes assume distinct transcriptional programming in patients with elevated inflammatory cytokines.

**Figure 4:**
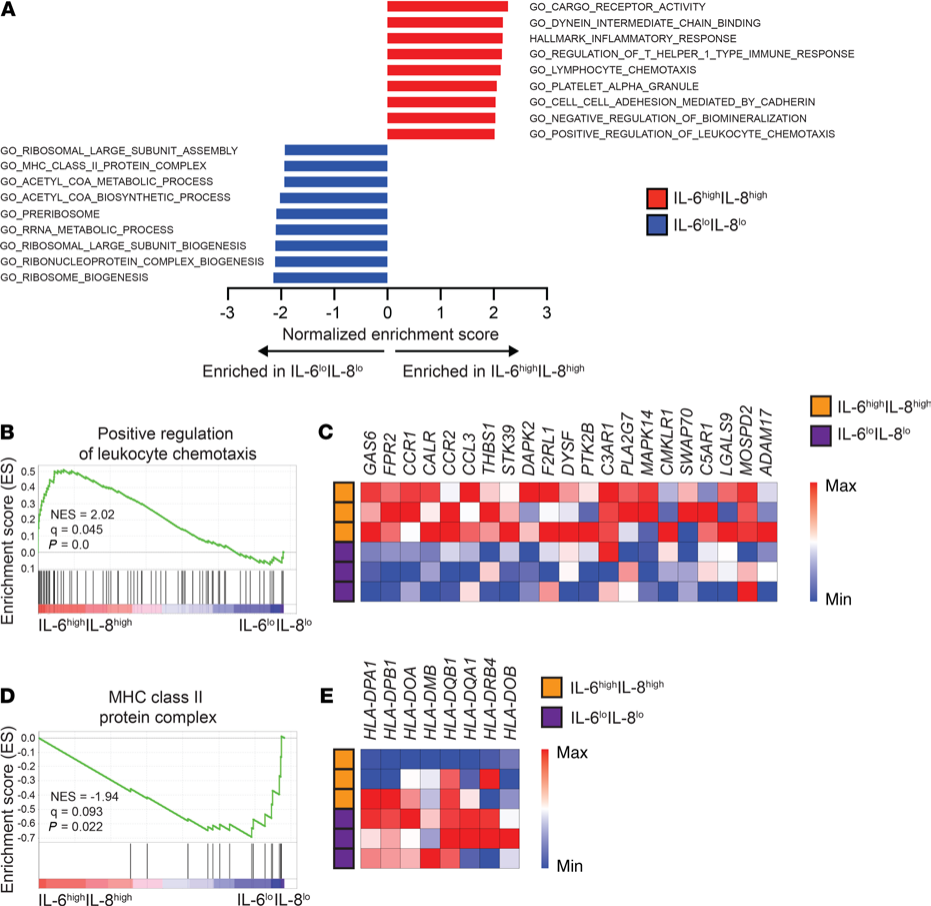
Circulating monocytes in patients with elevated inflammatory cytokines display distinct transcriptional programming. (**A**) Top gene sets enriched in monocytes from patients (*n* = 6) with high (IL-6^hi^IL-8^hi^) or low (IL-6^lo^IL-8^lo^) plasma IL-6 (cutoff 10 pg/mL) and IL-8 (cutoff 45 pg/mL). (**B**) Enrichment plot of positive regulation of leukocyte chemotaxis from gene set enrichment analysis (GSEA) shown in **A**. (**C**) Heatmap of selected genes from the positive regulation of leukocyte chemotaxis gene set enriched in IL-6^hi^IL-8^hi^ monocytes. (**D**) Enrichment plot of MHC class II protein complex from GSEA shown in **A**. (**E**) Heatmap of selected genes from the MHC class II protein complex gene set enriched in IL-6^lo^IL-8^lo^ monocytes. NES, normalized enrichment score; Gas6, growth arrest specific 6.

Inflammatory monocytes and macrophages play an important role in PDA-associated immunosuppression (38, 39). To understand phenotypic changes in myeloid cell biology and associations with potential cytokine drivers, we examined whether the transcriptional state of circulating monocytes was distinct in the presence of IL-6 and IL-8. To test this, we isolated CD14^+^ monocytes (Supplemental Figure 4A) from the peripheral blood of patients (*n* = 6) with high or low plasma cytokines (pCytokine^lo^ vs. pCytokine^hi^), based on IL-6 (cutoff 10 pg/mL) and IL-8 (cutoff 45 pg/mL) levels, and performed transcriptional profiling using a gene microarray approach. We found 90 differentially expressed genes (DEGs) among pCytokine^hi^ and pCytokine^lo^ monocytes with 89 genes differentially upregulated in pCytokine^hi^ monocytes (Supplemental Figure 4B). Notably, *CCR2*, which is an established marker of inflammatory monocytes in PDA (40), was upregulated in pCytokine^hi^ monocytes. Additionally, we found enrichment of inflammation-related gene sets, specifically response to inflammation and positive regulation of leukocyte chemotaxis in pCytokine^hi^ monocytes (Figure 4, A and B). DEGs enriched in the positive regulation of the leukocyte chemotaxis gene set included *CCR2*, *GAS6*, *formyl peptide receptor 2* (*FPR2*), and *thrombospondin 1* (*THBS1*) (Figure 4C). In contrast, gene sets enriched in pCytokine^lo^ monocytes included ribosomal biogenesis, acetyl CoA metabolism, and MHC class II protein complex (Figure 4, D and E). Taken together, these data show that monocytes in the peripheral blood assume a distinct transcriptional program in the presence of inflammatory cytokines.

### The kinetics of peripheral blood inflammatory markers suggest distinct responses to CD40-based chemotherapy among patients with systemic inflammation.

**Figure 5:**
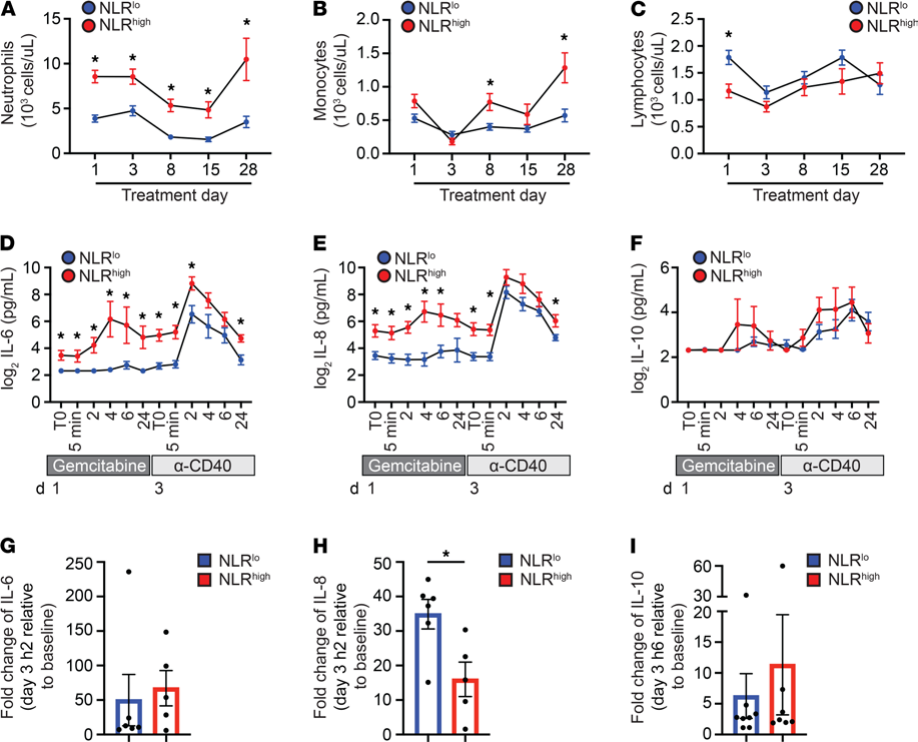
Interplay of chemoimmunotherapy and peripheral blood inflammatory markers. (**A**) Absolute neutrophil counts, (**B**) absolute monocyte counts, and (**C**) absolute lymphocyte counts in the peripheral blood over 1 cycle of treatment with gemcitabine and anti-CD40 therapy (*n* = 22). Patients stratified by baseline NLR as NLR^lo^ (NLR < 3.1, blue) or NLR^hi^ (NLR > 3.1, red). (**D**) Log_2_-transformed IL-6, (**E**) log_2_-transformed IL-8, and (**F**) log_2_-transformed IL-10 plasma levels at baseline, 5 minutes, 2 hours, 4 hours, 6 hours, and 24 hours after treatment consecutively with gemcitabine and anti-CD40 therapy (*n* = 18). Mean ± SEM is shown. Multiple *t* tests with correction of Benjamini and Hochberg with a FDR < 0.05 were performed. (**G**–**I**) The peak change in each cytokine was calculated. (**G**) IL-6 (*n* = 11) and (**H**) IL-8 (*n* = 11) levels were calculated as day 3, hour 2, relative to baseline (T0). (**I**) IL-10 (*n* = 15) levels were calculated as day 3, hour 6, relative to T0. Mann-Whitney *U* tests were performed. *P < 0.05.

We next evaluated treatment-associated changes in inflammatory markers among NLR^hi^ and NLR^lo^ patients. We first studied cellular dynamics in the peripheral blood based on clinical blood counts. In both groups, neutrophils decreased on treatment days 8 and 15. However, neutrophils were significantly higher in NLR^hi^ patients at all time points of cycle 1 (Figure 5A). Monocytes were also found to decrease on treatment day 3, after gemcitabine administration. Additionally, in NLR^hi^ patients, monocytes recovered to levels significantly higher than seen in NLR^lo^ patients on day 8 and remained significantly elevated at the end of cycle 1 (Figure 5B). Lymphocytes were significantly higher in NLR^lo^ patients at baseline but became similar among the groups during treatment (Figure 5C). Given these changes in neutrophils and lymphocytes with treatment, we next analyzed the dynamics of NLR after beginning treatment (Supplemental Figure 5A). Over 1 cycle of treatment, NLR remained significantly higher in the NLR^hi^ group as compared with the NLR^lo^ group (Supplemental Figure 5A). Additionally, in both groups, there was a transient decrease in NLR at day 15 after treatment, which coincided with a treatment-related decrease in neutrophils. At the end of 1 cycle of treatment, NLR in all NLR^hi^ patients remained greater than 3.1, while in NLR^lo^ patients, 3 of 8 patients had converted to a NLR greater than 3.1. We also assessed whether there were differences in the pharmacodynamic response of FlowSOM-defined clusters (≥1% of CD45^+^ cells) from the CyTOF data set among NLR^hi^ and NLR^lo^ patients. This analysis was limited by small numbers of patients in each group and heterogeneity in cluster frequency. However, changes in FlowSOM-defined clusters were largely similar among the groups (Supplemental Figure 5, B–J). Interestingly, transient B cell depletion, which is characteristic of a CD40 agonist, appeared to recover more rapidly to baseline by day 15 in NLR^lo^ patients, whereas NLR^hi^ patients continued to have B cell frequencies significantly lower than baseline on day 15 (Supplemental Figure 5H).

Furthermore, we assessed for acute changes in inflammatory cytokines by analyzing patient plasma collected pretreatment and between 5 minutes and 24 hours after treatment with gemcitabine or anti-CD40 mAb therapy. While modest changes in IL-6, IL-8, and IL-10 plasma levels were observed after gemcitabine administration, anti-CD40 mAb therapy was associated with significant increases in plasma concentrations of IL-6, IL-8, and IL-10 with a peak at 2–6 hours after treatment. Notably, baseline and peak IL-6 levels were highest in NLR^hi^ patients (Figure 5D). In contrast, although baseline IL-8 levels were higher in NLR^hi^ patients, peak IL-8 levels were similar among the 2 groups (Figure 5E). There was no difference in IL-10 plasma levels between NLR^hi^ and NLR^lo^ patients (Figure 5F). Additionally, the fold change in inflammatory cytokine concentration (peak relative to baseline) was different among NLR^hi^ and NLR^lo^ patients. For example, while there was no difference in fold change for IL-6 or IL-10, there was a significantly higher fold change in plasma IL-8 in NLR^lo^ patients as compared with NLR^hi^ patients (Figure 5, G–I). Taken together, these data show that distinct cellular and cytokine pharmacodynamics are present in NLR^hi^ and NLR^lo^ patients after treatment with chemoimmunotherapy.

### NLR defines patients with distinct outcomes of CD40-based chemoimmunotherapy.

**Figure 6:**
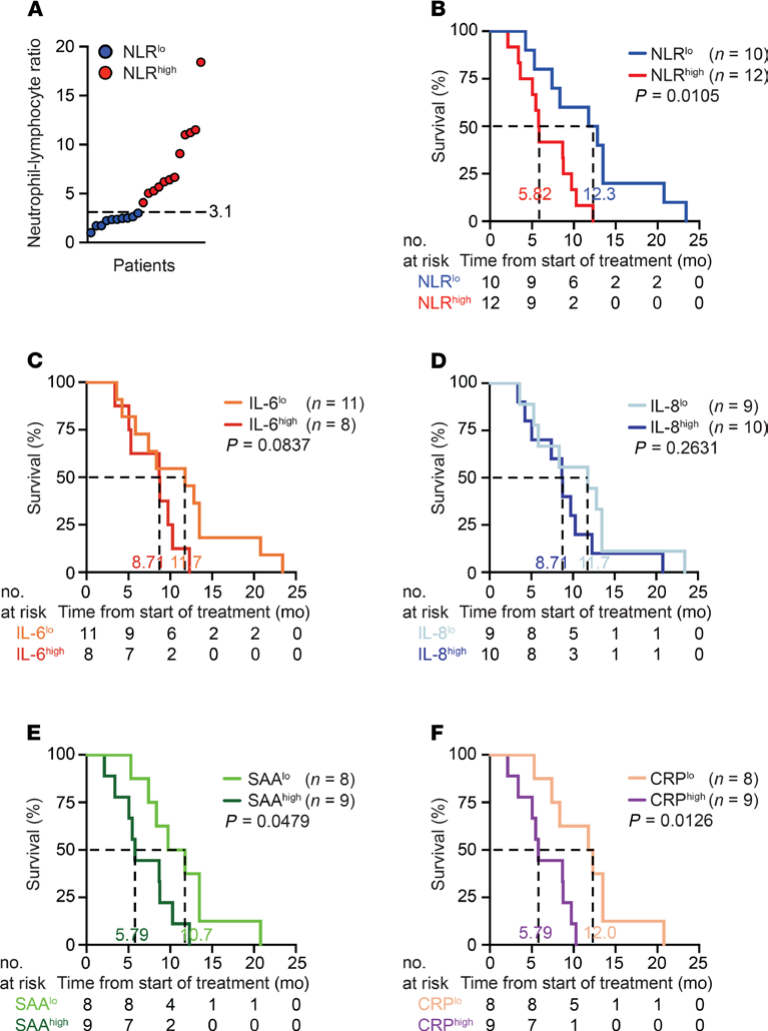
Elevated NLR is associated with poor outcomes in patients with pancreatic ductal adenocarcinoma treated with CD40 agonist–based chemoimmunotherapy. (**A**) Baseline NLR of individual patients. Dashed line indicates NLR cutoff of 3.1. Patients with NLR > 3.1 were designated as systemically inflamed (NLR^hi^) and patients with NLR < 3.1 were designated as noninflamed (NLR^lo^). (**B**) OS was estimated by Kaplan-Meier methodology and log-rank test was used to determine significance. (**C**–**F**) Univariate survival analysis using plasma (**D**) IL-6 median cutoff of 5 pg/mL, (**D**) IL-8 median cutoff of 14 pg/mL, (**E**) SAA median cutoff of 130 μg/mL, and (**F**) CRP median cutoff of 14.3 mg/L. Numbers indicate median OS.

Finally, we assessed clinical outcomes of chemoimmunotherapy among the 2 groups. Of the 22 patients included in our study, 12 patients were NLR^hi^ and 10 patients were NLR^lo^ (Figure 6A). Patient characteristics were well balanced, although more NLR^hi^ patients had liver metastases (100% vs. 70%) and more NLR^lo^ patients had peritoneal metastases (30% vs. 0%) (Supplemental Table 1). In a univariate analysis, OS was significantly shorter in NLR^hi^ patients as compared with NLR^lo^ patients (5.82 vs. 12.3 months; *P* = 0.0105) (Figure 6B). Additionally, we performed a multivariate analysis including sex, Eastern Cooperative Oncology Group (ECOG) performance status, tumor burden, and age and found NLR more than 3.1 continued to correlate with worse OS (HR 3.87; CI 1.04–14.38; *P* = 0.043) (Supplemental Figure 6A). Intriguingly, when patients were dichotomized using the median of acute phase reactants (SAA and CRP) and inflammatory cytokines (IL-6 and IL-8), only elevated acute phase reactants, not inflammatory cytokines, were significantly associated with poor OS (Figure 6, C–F).

## Discussion

In this study, we used high-dimensional cellular phenotyping and plasma cytokine analysis to evaluate the immune response to a CD40 agonist in combination with gemcitabine chemotherapy in the peripheral blood of patients with advanced PDA. Notably, CD40-based chemoimmunotherapy was associated with transient activation of CD4^+^ T cells and changes in monocytes and B cells. However, T cell activation in response to therapy was not associated with outcomes. In contrast, the presence of a preexisting systemic inflammatory response was found to associate with reduced survival. Taken together, our data suggest that although a CD40 agonist can induce T cell activation in patients, additional determinants of response exist. Furthermore, our findings identify systemic inflammation as a potential resistance mechanism to CD40-based chemoimmunotherapy.

One limitation of our study is the choice of chemotherapy. At the time of study initiation, gemcitabine was the only US Food and Drug Administration–approved systemic therapy for the treatment of advanced PDA. Currently, 5-fluorouracil in combination with oxaliplatin and irinotecan (FOLFIRINOX) and gemcitabine combined with nab-paclitaxel are standard of care (41, 42). It is uncertain if multiagent chemotherapy as compared with gemcitabine alone in combination with a CD40 agonist might generate distinct immune responses, as has been suggested in preclinical models (20, 43). In this regard, an ongoing phase Ib/II trial (ClinicalTrials.gov NCT03214250), studying the combination of a CD40 agonist (APX005M) and gemcitabine plus nab-paclitaxel with or without nivolumab, will be informative (18).

Another limitation of our study is the single-arm design, which limits definitive conclusions regarding efficacy measures. However, a subset analysis of the MPACT phase III trial, which examined NLR as a determinant of outcomes in gemcitabine plus nab-paclitaxel versus gemcitabine monotherapy, provides some context for our findings (44). In this study, an NLR cutoff of 5 was used. For patients with NLR less than 5, treatment with gemcitabine plus nab-paclitaxel compared with gemcitabine monotherapy was associated with median OS of 10.9 and 7.9 months, respectively. In contrast, median OS for patients with NLR more than 5 was 5.6 months for gemcitabine plus nab-paclitaxel and 4.3 months for gemcitabine monotherapy. Although we used a lower NLR cutoff in our study, we have also examined survival outcomes based on an NLR of 5 (Supplemental Table 2). We found that median OS was 11.7 months (NLR < 5) and 5.8 months (NLR > 5) for CD40-based chemoimmunotherapy, which compares favorably and suggests that CD40-based treatment may be most effective in patients with a low NLR.

Cytotoxic chemotherapy can have both immunosuppressive and immune-stimulating capacity (45). Importantly, the optimal sequencing of chemotherapy in combination with CD40-based immunotherapy remains ill-defined. In our study, we found near complete depletion of monocytes and a CD56^+^CD11c^+^HLA-DR^+^CD141^+^ population in the peripheral blood after chemotherapy administration, which was transient but persisted through the day of CD40 agonist treatment. These findings are consistent with those of others who have shown gemcitabine induces transient decreases in monocytes, DC precursors, and T regulatory cells, while largely having no impact on B and T cell frequency or phenotype (46, 47). Notably, both monocytes and DCs are important in the mechanism of action of a CD40 agonist (20, 22). Thus, chemotherapy, when delivered prior to anti-CD40 therapy, may compromise the full activity of treatment. In contrast, administration of anti-CD40 therapy prior to chemotherapy may leverage the antistromal effects of a CD40 agonist, thereby potentiating the activity of chemotherapy (22, 23). To this end, treatment with a CD40 agonist delivered at least 4 days prior to chemotherapy is safe and produces promising antitumor activity in mouse models of PDA (23). However, the timing of chemotherapy treatment is critical, as delivering a CD40 agonist within 3 days prior to chemotherapy can trigger lethal hepatotoxicity in mice (23, 48). An alternative strategy that remains unexplored clinically is whether CD40-based immunotherapy might provide benefit in the maintenance setting after induction chemotherapy. Maintenance immunotherapy with checkpoint inhibition was recently established in the JAVELIN-100 trial, which showed improved survival in patients with advanced bladder cancer treated with maintenance anti–PD-L1 therapy following induction chemotherapy (49). Our results suggest further study is warranted to determine the optimal sequencing of anti-CD40 therapy and chemotherapy.

Preclinical evidence shows that a CD40 agonist, especially when combined with checkpoint inhibition, leads to CD4^+^ T cell–mediated antitumor immune responses (50, 51). We observed a CD4^+^ T cell response in the peripheral blood of patients following treatment with CD40-based chemoimmunotherapy, providing evidence that this biology can also be observed in humans. Intriguingly, bona fide cytotoxic CD4^+^ T cells have been described in patients with bladder cancer and when present intratumorally are associated with improved responses to checkpoint inhibition (52). Additionally, we saw no consistent evidence of CD8^+^ T cell activation. In mouse models of PDA, both CD8^+^ and CD4^+^ T cells are required for the activity of CD40-based chemoimmunotherapy (21). It remains possible that absence of CD8^+^ T cell response limits the full therapeutic potential of CD40-based treatment and contributes to the lack of association between cellular pharmacodynamics and outcomes.

One limitation to our study is that tissue biopsies were not available for analysis and we cannot confirm if peripheral blood immune dynamics are representative of responses occurring in secondary lymphoid organs or tumor. To this end, preclinical models show that a CD40 agonist can trigger systemic T cell responses without affecting the intratumor T cell compartment (21, 22). However, activation of circulating monocytes by a CD40 agonist is correlated with myeloid cell activation within tumors (23). Moreover, CD40-activated monocytes are functionally important and can sensitize tumors to chemotherapy (23). Additionally, others have shown an association between peripheral blood leukocyte composition and outcomes in patients with PDA. For example, the presence and diversity of peripheral blood T cells reactive against the tumor-associated antigen mesothelin is associated with prolonged disease-free survival in patients with PDA treated with immunotherapy (53, 54). Taken together, these data highlight the potential of peripheral blood leukocyte changes to associate with immune cell dynamics in the TME and correlate with clinical outcomes.

Inflammatory monocytes and tumor-associated macrophages are intimately associated with PDA resistance to productive T cell immunosurveillance (55). Consistent with the reports of others, we found monocytes to be elevated in patients with poor outcomes (40). Moreover, we identified upregulation of *CCR2* and *GAS6* in CD14^+^ monocytes from patients with elevated plasma levels of inflammatory cytokines. One potential limitation of our approach is that we evaluated monocytes in patients defined by plasma cytokine levels rather than NLR. Nonetheless, our findings provide insight into associations among specific inflammatory cytokines and monocyte phenotype. To this end, targeting of CCR2^+^ macrophages using CCR2 inhibitors is an effective method of tumor control in mouse models of PDA and has shown safety and potential clinical activity in combination with FOLFIRINOX in patients (40, 56). However, we have also shown that CCR2 inhibition can impair the capacity of a CD40 agonist to improve the efficacy of chemotherapy in mouse models of PDA (23). Gas6, which is an AXL kinase ligand, may be an alternative target. Notably, Gas6 has been implicated in PDA tumor progression (57, 58). Our findings suggest that inflammatory monocytes may be a source of GAS6. Furthermore, blockade of AXL has shown promise in preventing PDA tumor growth (57).

In addition to monocytes and macrophages, neutrophils are an important determinant of cancer biology. Neutrophils play a pleiotropic role in cancer and can enact both pro- and antitumor activity depending on features of the TME (59). Further, monocyte-depleting therapies can trigger a compensatory increase in immunosuppressive tumor-associated neutrophils (60). Mouse models of PDA show that tumor-associated neutrophils are recruited to the TME via the CXCR2/ligand axis and can coordinate immunosuppression and limit T cell infiltration into tumors (61). In this regard, tumor-derived CXCL1, a ligand for CXCR2, has been implicated as a mechanism of resistance to CD40 immunotherapy (62). Intriguingly, dual targeting of CXCR2^+^ neutrophils and CCR2^+^ macrophages also prevents reciprocal increases in immunosuppressive myeloid cells and facilitates T cell immunosurveillance in mouse models of PDA (60). Taken together, these findings highlight the potential for incorporating blockade of inflammatory myeloid cells into CD40-based treatment regimens to improve outcomes in the setting of systemic inflammation.

Accumulating evidence suggests that systemic inflammation is a mechanism of resistance to immunosurveillance, rather than simply a surrogate of aggressive cancer biology. To this end, soluble factors (e.g., CRP, SAA, IL-6, IL-8) directly influence innate and adaptive immunity, highlighting the immunosuppressive functionality of components of the systemic inflammatory response (11, 63–65). The biological activity of a CD40 agonist may be especially susceptible to the immunomodulatory effects of inflammatory factors. For example, CRP, SAA, and IL-6 can each influence the biology of DCs, the purported key cellular target of CD40 antibodies, by inhibiting maturation and driving apoptosis (11, 63, 64, 66). Inflammatory factors can also drive DC dysfunction. For example, activation of TLR2 on DCs leads to increased sensitivity to IL-6 signaling, which subsequently triggers development of an immunosuppressive DC phenotype (67).

Thus, systemic inflammation encompasses the activity of a network of factors. Given the complexity of systemic inflammation, it remains likely that many of these inflammatory components are nonredundant and will need to be individually targeted. Additionally, further investigation into mechanisms by which inflammatory cues mediate the fate of DCs will be needed to inform therapeutic strategies that reprogram DCs in the setting of systemic inflammation.

In our study, we found that elevated levels of IL-6 and IL-8 in the blood at baseline were associated with shortened survival. Notably, systemic CD40 activation also triggered transient elevations in IL-6 and IL-8. It remains unclear whether these acute changes in inflammatory cytokines are beneficial or detrimental to anti-CD40 efficacy. In preclinical studies, IL-6 blockade produced no impact on the antitumor activity of a CD40 agonist (68). Furthermore, anti–IL-6 therapy can enhance the activity of anti–PD-1 treatment in mouse models of PDA (69). However, in contrast to acute changes in inflammatory cytokines produced by CD40 treatment, chronic expression can lead to a deficiency in DCs important for T cell immunity (11), impairment in the efficacy of chemotherapy (70), and enhanced metastatic risk (34). Taken together, these observations provide rationale for testing the contributions of distinct cytokines to the activity of CD40-based treatments.

Immunotherapy has thus far failed to improve outcomes for patients with PDA. However, myeloid targeted immunotherapy is a distinct treatment approach that has shown promise. In this study, we examined the activity of a CD40 agonist, which can drive innate and adaptive immunity. Unexpectedly, we saw no consistent evidence of CD8^+^ T cell activation, and CD4^+^ T cell activation did not correlate with outcomes. Furthermore, our data suggest chemotherapy may have a detrimental impact by eliminating monocytes and DCs, which are cells that are fundamental to facilitating T cell–dependent immune responses. Thus, non–T cell–based mechanisms may govern the therapeutic activity of systemic CD40 activation in combination with gemcitabine. Our data also suggest that acute phase reactants (SAA and CRP) and monocyte transcriptional programming may be determinants of response to CD40-based treatment. Overall, our study provides insight into the cellular and biological mechanisms of response and resistance to a CD40 agonist combined with chemotherapy in patients with advanced PDA.

## Methods

### Patients, clinical samples, and clinical data collection.

Samples for this analysis were collected from HVs recruited at the University of Pennsylvania and from a previously completed phase I clinical study investigating the combination of gemcitabine and CP-870,893 (anti-CD40 mAb) for the treatment of patients (*n* = 22) with advanced PDA (17). Clinical data including demographics and characteristics and clinical laboratory tests were extracted from the electronic medical record. NLR, WBC, ANC, ALC, ABC, AEC, AMC, platelets, and albumin level were based on clinical chemistry and hematology lab analysis. Patients were defined as being noninflamed (NLR^lo^) or systemically inflamed (NLR^hi^) based on pretreatment NLR with cutoffs of greater or less than 3.1. The cutoff was chosen based on a prior study that identified NLR as a prognostic marker in patients with advanced PDA (32).

### PBMC collection and isolation.

Whole blood was collected in sodium heparin or EDTA tubes and centrifuged at 1200 rpm for 10 minutes at room temperature with plasma removed afterward. Blood was diluted with RPMI medium and layered on Ficoll, then centrifuged at 2200 rpm for 20 minutes at room temperature. Interphase containing PBMCs was removed, washed, and cryopreserved in liquid nitrogen until analysis.

### Detection of cytokines, SAA, and CRP.

Plasma was collected and stored at –80°C until analysis. Cytokine levels (IL-2, IL-4, IL-5, IL-1b, IL-6, IL-8, IL-10, IL-12, IFN-γ, TNF) and SAA levels were determined using human ELISA kits (Invitrogen, Thermo Fisher Scientific). CRP levels were determined by Cobas c311 assay (Roche).

### Monocyte isolation, gene array, differential gene expression analysis, and pathway analysis.

Cryopreserved PBMCs were thawed and counted using a Z2 Coulter Counter Analyzer (Beckman Coulter). CD14^+^ cells were isolated using positive selection with human CD14 microbeads (Miltenyi Biotec). Cell purity was assessed by flow cytometry using commercially available antibodies (CD14-APC-Cy7; clone MφP9; catalog 557831; BD Pharmingen) and was routinely greater than 95% (Supplemental Figure 4A). Isolated CD14^+^ cells were processed with TRIzol treatment for RNA isolation (Thermo Fisher Scientific), and RNA was submitted to the Wistar Institute. RNA quality was assessed using 2100 Bioanalyzer (Agilent), and samples were analyzed using a HumanHT-12 v4 BeadChip (Illumina). Significance analysis of microarray was used to determine differential gene expression analysis with a FDR of 0.2. Pathway and gene ontology analysis were performed using GSEA (GO and HALLMARK gene sets) with FDR of 0.25.

### Mass cytometry antibody panel, staining, and data acquisition.

Mass cytometry staining and data acquisition were performed as previously described (11). See Supplemental Table 3 for antibody panel information. In brief, PBMCs were thawed and washed with FACS buffer. Then 4 × 10^6^ or fewer cells per patient were stained with live/dead (1 μM 198PT monoisotopic cisplatin; Fluidigm). Cells were incubated in Cytofix fixation buffer, washed, and barcoded using palladium metal barcodes as per the manufacturer’s instructions (Fluidigm). Cells were incubated with Human TruStain FcX (BioLegend) and stained with an antibody master mix for 30 minutes at room temperature. After washing, cells were fixed with 2.4% formaldehyde in PBS containing 125 nM iridium nucleic acid intercalator (Fluidigm) and kept overnight. Cells were cryopreserved and stored at –80°C until thawing for acquisition. Cells were washed and resuspended at a concentration of 1 × 10^6^ cells/mL in cell acquisition solution with 5% EQ beads (Fluidigm). Acquisition was performed using a Helios mass cytometer (Fluidigm) and a standardized acquisition template. FCS files were bead-normalized and debarcoded using Helios software (Fluidigm). Using FlowJo (BD), debris, dead cells, and doublets were excluded.

### Statistics.

OS was defined as the number of days from start of treatment on trial to the date of death from any cause. Kaplan-Meier methodology was used to assess OS in univariate analyses stratified by NLR with a cutoff of 3.1, IL-6 with a median cutoff of 5 pg/mL, IL-8 with a cutoff of 14 pg/mL, SAA with a cutoff of 130 μg/mL, and CRP with a cutoff of 14.3 mg/L. Log-rank (Mantel-Cox) test was used to compare the OS between groups. To account for potential differences in baseline characteristics when assessing the interaction between NLR and OS, we conducted a Cox proportional hazard model of OS adjusting for age, sex, ECOG performance status, and tumor burden, which was defined as the sum of target lesions. Mann-Whitney *U* tests and Wilcoxon’s tests were used for comparison of unpaired and paired continuous variables, respectively. Fisher’s test was used for comparison of categorical variables. All tests were performed using a 2-sided α of 0.05. Spearman’s correlation coefficients were calculated to quantify correlations between features. Correlations were visualized using correlograms generated with the R function *corrplot* and showing positive correlations in blue and negative correlations in red when *P* < 0.05. Density plots were generated using the kde2d function from the MASS package in R. Where appropriate, multiple-comparison testing was performed using the FDR correction of Benjamini and Hochberg with FDR < 0.05. Mixed effects analysis with Dunnett’s multiple-comparison test was used to compare changes in cellular pharmacodynamics. One-way ANOVA with Tukey’s correction for multiple comparisons was used when multiple groups were compared. Statistical analysis was performed using Prism 8.0 software (GraphPad) and R.

### Study approval.

All participants or their surrogates provided written informed consent in accordance with protocols approved by the Institutional Review Board of the University of Pennsylvania and the Declaration of Helsinki. The protocol was approved by the institutional review board of the University of Pennsylvania.

## Author contributions

MMW and GLB designed the experiments. MMW, VMH, MAG, and WLG performed the experiments. MMW, VMH, WLG, and MAG analyzed the data. MMW wrote the manuscript. MMW, VMH, WLG, MAG, ELC, and GLB reviewed and edited the manuscript.

## Supplementary Material

Supplemental data

## Version 1 01/26/2021

In-Press Preview

## Version 2 03/08/2021

Electronic publication
