# Supplementary material for: Systemic inflammation is a determinant of outcomes of CD40 agonist–based therapy in pancreatic cancer patients
Source: JCI Insight. 2021 Mar 8;6(5):e145389. doi: 10.1172/jci.insight.145389 (PMC8021099; doi:10.1172/jci.insight.145389)
Supplement: Supplemental data [file jciinsight-6-145389-s116.pdf]

Supplementary Table S1. Demographic and baseline characteristics			
**Characteristic**	**NLR^{low} (*n* = 10)**	**NLR^{high} (*n* = 12)**	**P value**
Age at diagnosis - yr			
Median	58	59	0.55
Range	40-72	51-81	-
Sex - no. (%)			
Male	8 (80)	6 (50)	0.2
Female	2 (20)	6 (50)	0.2
Race or ethnic group - no. (%)			
White	8 (80)	11 (92)	0.57
Black	1 (10)	1 (8)	>0.9
Asian	1 (10)	0 (0)	0.45
ECOG - no. (%)			
0	5 (50)	4 (33)	0.67
1	5 (50)	8 (66)	0.67
Extent of Disease - no. (%)			
Locally Advanced	2 (20)	0 (0)	0.19
Metastatic	8 (80)	12 (100)	0.19
Location of Primary - no (%)			
Head	2 (20)	6 (50)	0.2
Body or Tail	7 (70)	4 (33)	0.2
Unknown	1 (10)	2 (16)	>0.9
Site of Metastases - no. (%)			
Liver	7 (70)	12 (100)	0.078
Lung	0 (0)	1 (8)	>0.9
Peritoneal	3 (30)	0 (0)	0.078
Bone	0 (0)	2 (17)	0.48
Other	1 (10)	1 (8)	>0.9
Prior Radiation Therapies - no. (%)			
No	10 (100)	11 (92)	>0.9
Yes	0 (0)	1 (8)	>0.9

Supplementary Table S2. Survival comparison				
**Study**	**Treatment**	***n***	**NLR cutoff**	**median OS**
Wattenberg et al	CD40 agonist and gemcitabine	10	< 5	11.7
		12	> 5	5.8
Goldestein et al	Gemcitabine plus nab-paclitaxel	266	< 5	10.9
		160	> 5	5.6
	Gemcitabine	277	< 5	7.9
		149	> 5	4.3

Supplementary Table S3. Antibody panel information				
**Antibody**	**Source**	**Product #**	**Tag**	**Clone**
CD196/CCR6	Fluidigm	3141014A	141Pr	11A9
CD11a	Fluidigm	3142006B	142Nd	HI111
CD123	Fluidigm	3143014B	143Nd	6H6
CD38	Fluidigm	3144014B	144Nd	HIT2
CD4	Fluidigm	3145001B	145Nd	RPAT4
CD64	Fluidigm	3146006B	146Nd	10.1
CD11c	Fluidigm	3147008B	147Sm	Bu15
CD16	Fluidigm	314800rB	148Nd	WM53
CD66a	Fluidigm	3149018B	149Sm	ASL32
MIP1beta	Fluidigm	3150004B	150Nd	D211351
LAMP1	Fluidigm	3151002B	151Eu	H4A3
TNFa	Fluidigm	3152002B	152Sm	Mab11
BDCA-2/CD303	Fluidigm	3153007B	153Eu	201A
CD163	Fluidigm	3154007B	154Sm	GHI/61
CD1b	Fluidigm	3155007B	155Gd	SN13
CD86	Fluidigm	3156008B	156Gd	IT2.2
CD169	Fluidigm	3158027B	158Gd	CD169
PD-L1	Fluidigm	3159029B	159Tb	29E.2A3
CD14	Fluidigm	3160001B	160Gd	M5E2
CD80	Fluidigm	3161023B	161Dy	2D10.4
CD8a	Fluidigm	3162015B	162Dy	RPAT8
CD33	Fluidigm	3163023B	163Dy	WM53
CD15	Fluidigm	3164001B	164Dy	W6D3
CD40	Fluidigm	3165005B	165Ho	5C3
CD34	Fluidigm	3166012B	166Er	581
CD1a	Fluidigm	3167012B	167Er	HI149
CD206	Fluidigm	3168008B	168Er	152
CD19	Fluidigm	3169011B	169Tm	HIB19
CD3	Fluidigm	3170001B	170Er	UCHT1
CXCR5	Fluidigm	3171014B	171Yb	RF8B2
CX3CR1	Fluidigm	3172017B	172Yb	2A91
CD141	Fluidigm	3173002B	173Yb	1A4
HLA-DR	Fluidigm	3174001B	174Yb	L243
PD1	Fluidigm	3175008B	175Lu	EH12.2H7
CD56	Fluidigm	3176003B	176Yb	CMSSB
CD11b	Fluidigm	3209003B	209Bi	ICRF44
CD45	Fluidigm	3089003B	Y89	HI30

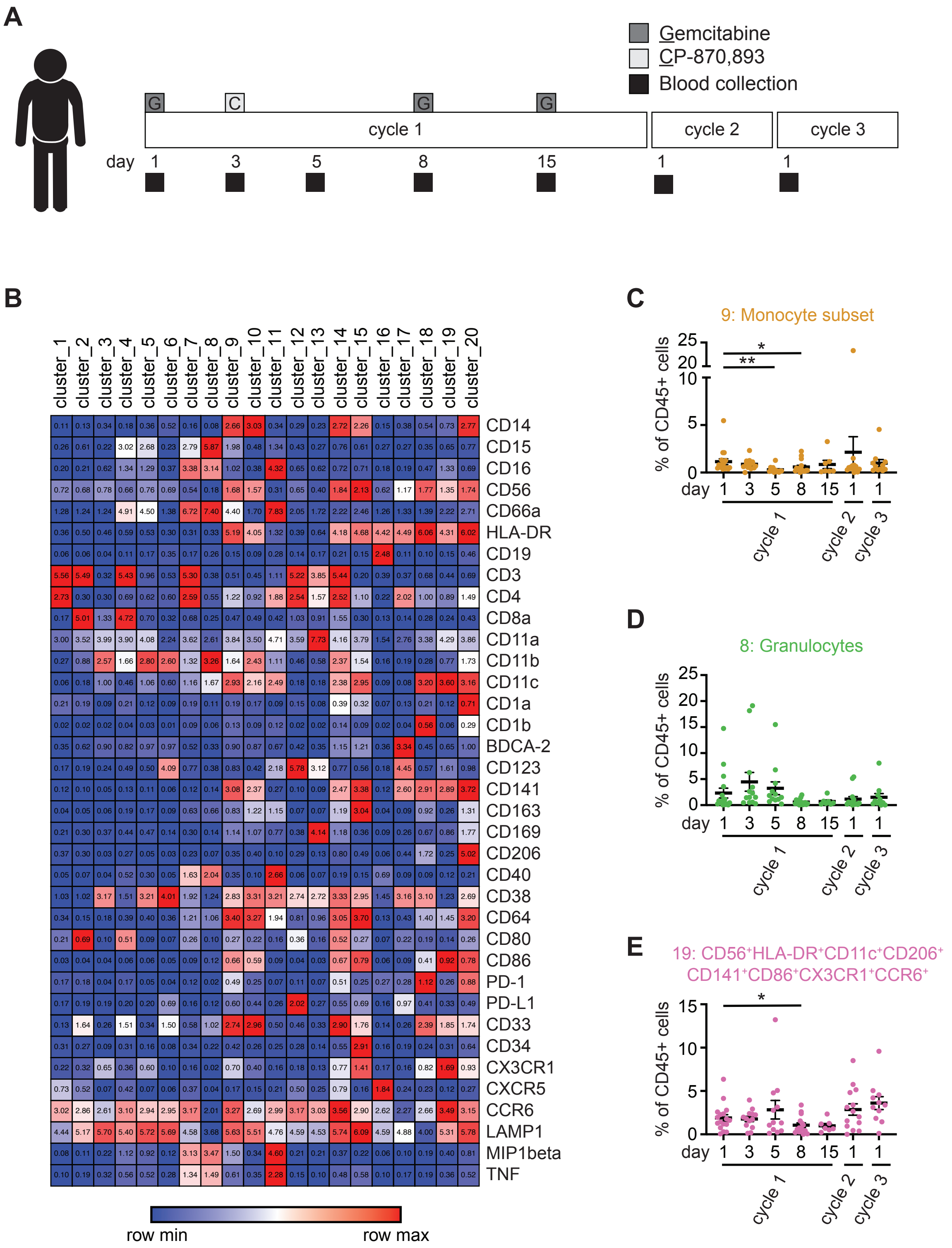

Supplementary Figure S1.

(A) Study design. Black boxes indicate blood collection. G, gemcitabine; C, CP-870-893. **(B)** Heatmap showing relative mean marker expression values of $CD45^+$ FlowSOM defined clusters. Patient and healthy volunteer samples were concatenated prior to analysis. **(C-E)** Quantification of cluster frequency. Mean ± SEM is shown. Day 1, n = 17; day 3, n = 13; day 5, n = 12; day 8, n = 15, day 15, n = 7, cycle 2, n = 14, cycle 3, n = 11. Mixed effects analysis with Dunnett's multiple comparison test was performed. *, $p < 0.05$; **, $p < 0.01$.

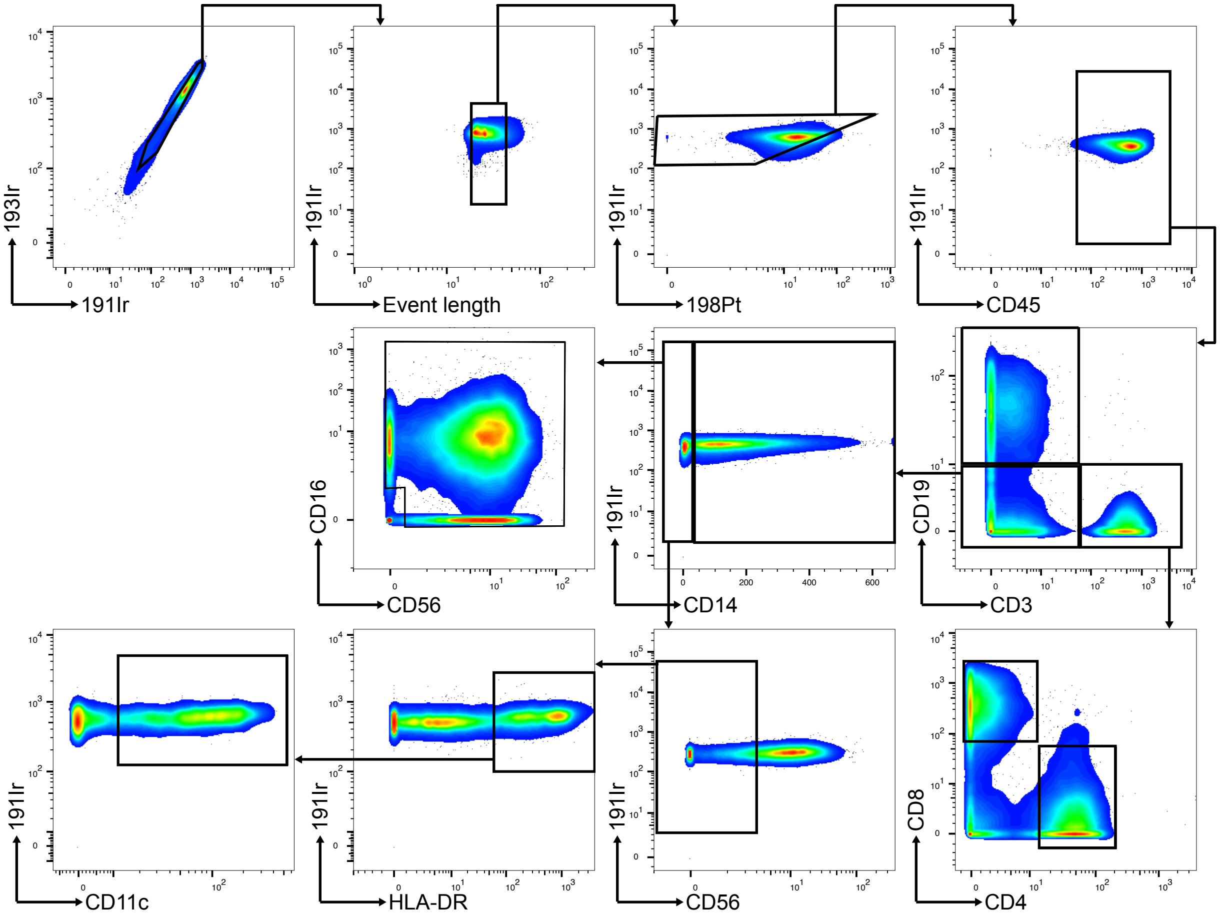

Supplementary Figure S2. Gating strategy

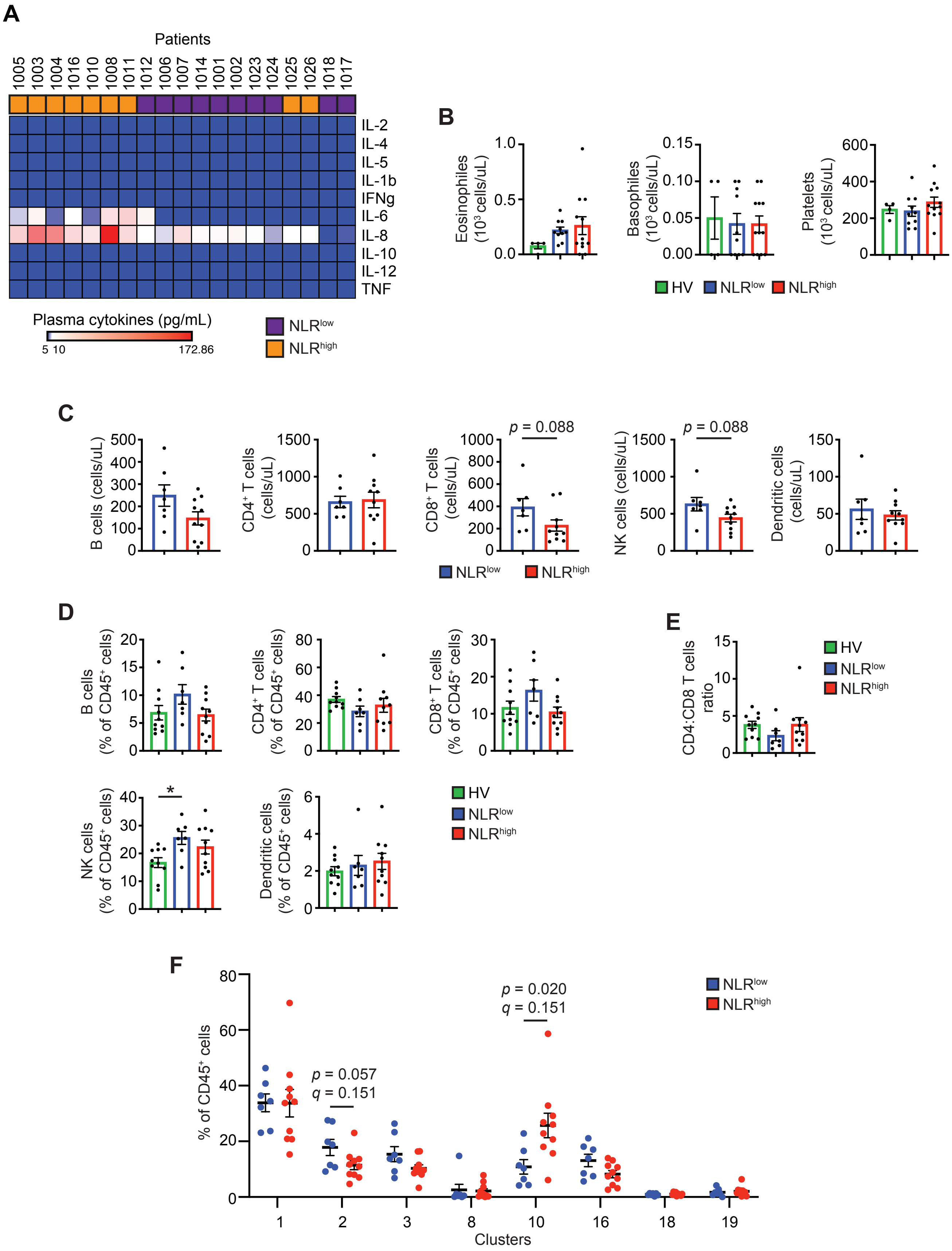

Supplementary Figure S3. | See next page for caption.

Supplementary Figure S3.

(A) Heatmap of plasma cytokines. Each column is an individual patient (n = 19). **(B)** Quantification of absolute eosinophil, basophil and platelet counts among HV (n = 4), NLR^{low} (n = 10) and NLR^{high} (n = 12) patients. **(C)** Quantification of absolute counts of immune cell populations among NLR^{high} (n = 10) and NLR^{low} (n = 7) patients. Absolute counts were calculated by multiplying the indicated cell subset (as a percentage of $CD45^+$ cells) times the total white blood cell count minus granulocytes. **(D)** Quantification of manually gated immune cell populations (as percentage of $CD45^+$ cells). **(E)** Quantification of CD4:CD8 T cell ratio. **(F)** Quantification of $CD45^+$ FlowSOM defined cluster frequencies which are $\geq$ 1% of $CD45^+$ cells. Clusters are defined as in **Supplementary Figure S1B**. Mean $\pm$ SEM is shown. ANOVA with Tukey's correction for multiple comparisons **(B, E)**, Mann-whitney tests **(C)** and Multiple t-tests were performed with Benjamini and Hochberg correction **(F)** were performed. *, $p < 0.05$. HV, healthy volunteer.

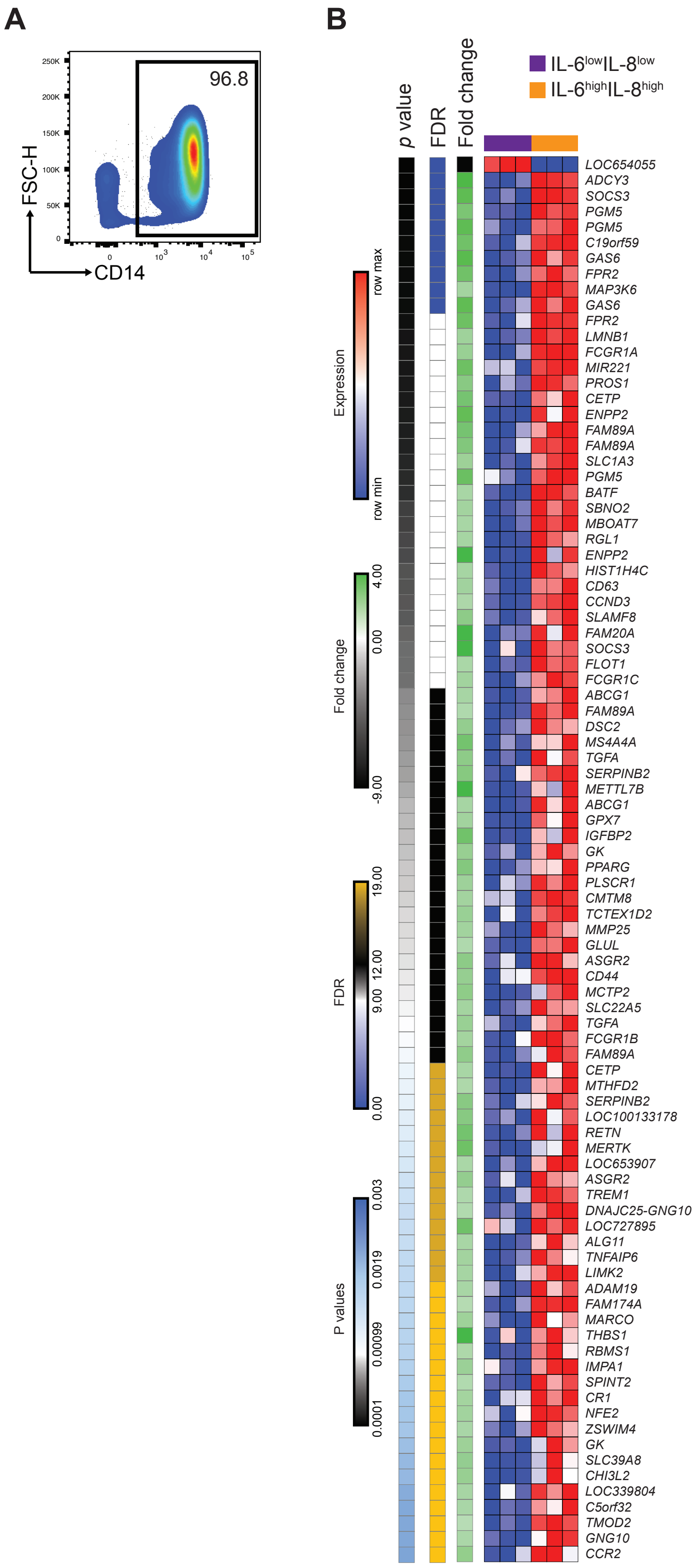

Supplementary Figure S4.
(A) Purity of bead selected CD14⁺ cells by flow cytometry. **(B)** Differential gene expression among monocytes from patients with low plasma cytokines (pCytokine^{low}; IL-6 < 10 pg/mL and IL-8 < 45 pg/mL) and high plasma cytokines (pCytokine^{high}; IL-6 > 10 pg/mL and IL-8 > 45 pg/mL). Significance analysis of microarray was used with FDR < 0.2.

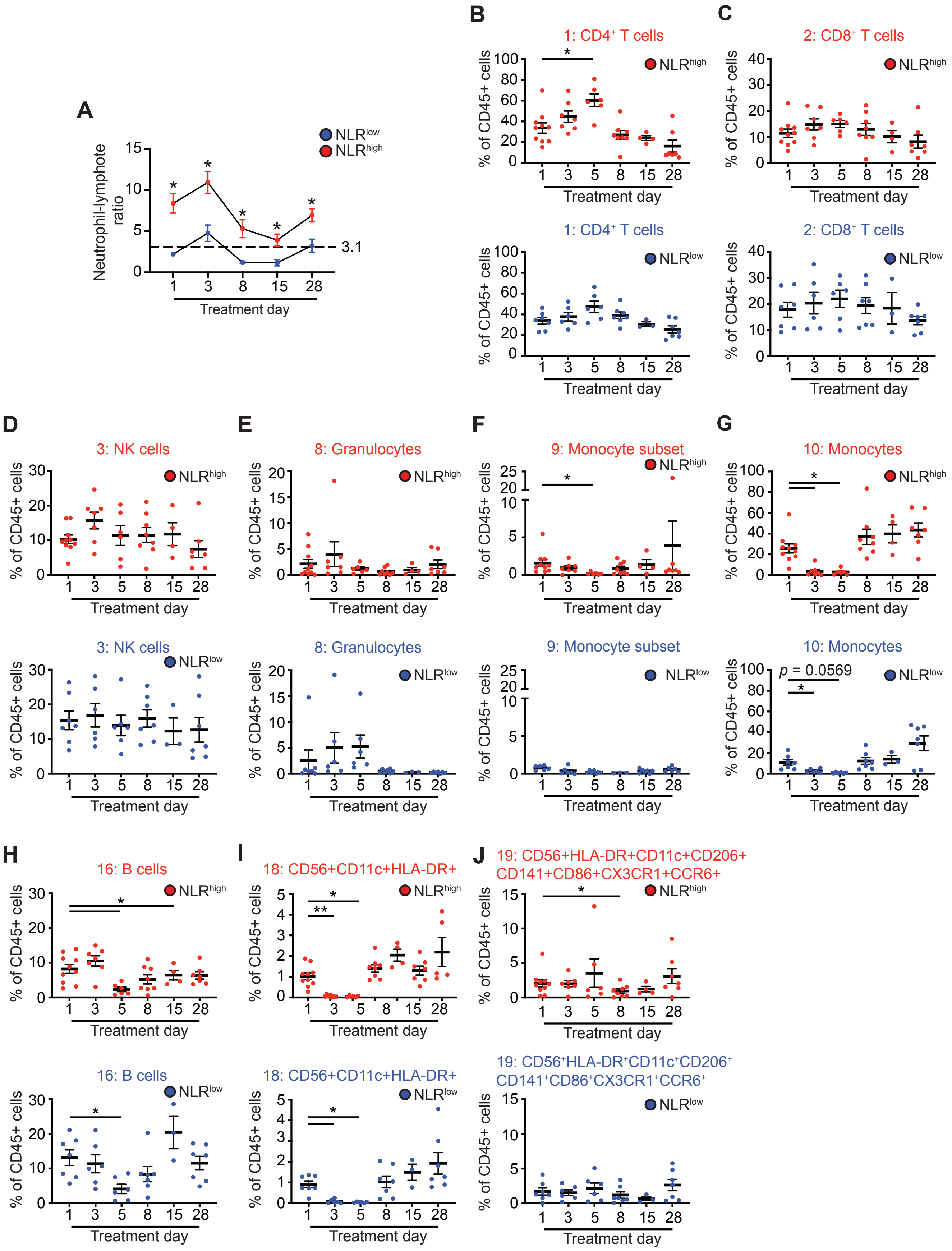

Supplementary Figure S5. | See next page for caption.

Supplementary Figure S5.

(A) Neutrophil-lymphocyte ratio (NLR) in the peripheral blood over one cycle of treatment with gemcitabine and anti-CD40 therapy (n = 22). Dashed line represents NLR cutoff. Patients stratified by baseline neutrophil-lymphocyte ratio (NLR) as NLR^{low} (NLR < 3.1, blue) or NLR^{high} (NLR > 3.1, red). Multiple t-tests were performed with Benjamini and Hochberg correction. **(B-J)** Quantification of cluster frequency. Clusters are defined as in **Supplementary Figure S1B**. Patients were defined by baseline NLR as NLR^{low} (<3.1) or NLR^{high} (>3.1). Mean ± SEM is shown. Day 1, n = 17; day 3, n = 13; day 5, n = 12; day 8, n = 15, day 15, n = 7, day 28, n = 14. Mixed effects analysis with Dunnett's multiple comparison test was performed. *, $p < 0.05$.

A

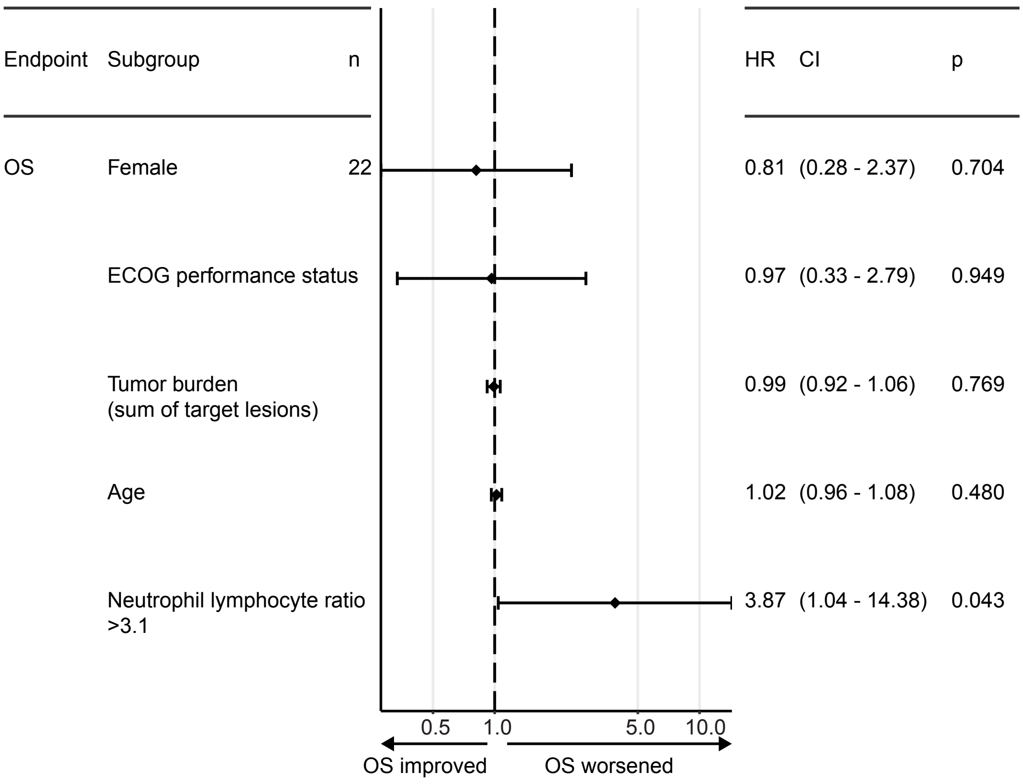

Supplementary Figure S6.
(A) Multivariate survival analysis using sex, age, ECOG performance status, tumor burden (sum of baseline RECIST measurements) and neutrophil-lymphocyte ratio greater or less than 3.1.
